# Report on the Medical Department of the University of Pennsylvania, for the Year 1847: To the Alumni of the School

**Published:** 1847-10

**Authors:** 

**Affiliations:** Philadelphia


### EDITORIAL DEPARTMENT.

*Report on the Medical Department of the University of Pennsylvania, for the year* 1847: *to the alumni of the school.* By the Medical Faculty, Philadelphia, 1847.

This report commends itself to the attention of the Medical Profession with peculiar force at the present time, from the fact that the Faculty therein declare their recognition of the obligation imposed upon them to endeavor to conform to the recommendations of the National Convention, and profess readiness to act in accordance with that acknowledged obligation. In conformity with these sentiments, as our readers are aware, the approaching session of lectures in that institution is to be extended to five months. But the interest which attaches to the report is not to be measured by this particular measure of medical reform. It is in view of the broad principle that in the National Association shall be vested legislative authority to prescribe measures designed to elevate the character and usefulness of the profession, medical schools being bound to carry out, as far as practicable, the spirit of these measures, that this document appears to us to posses not a little importance. The oldest, and, it may be added without invidiousness, the most respected medical school in the country has taken a position in favor of this principle. It is not derogating from the independence of other institutions to say, that the stand taken by the University of Pennsylvania, will exert not a little influence in bringing about a similar action by the schools generally. We do not hesitate individually to commit ourselves in advocacy of that principle, here and elsewhere, so long as the National Association shall con_tinue to represent the intelligence and wisdom of the Profession; and this will be the case so long as at its meetings are assembled members selected from among the respectable, well-informed Practitioners of different sections of the country. With such a body of men, the utmost confidence may be felt that every thing done will be done with deliberation and prudence, dictated by earnest, disinterested aims for proper objects, and calculated to secure their accomplishment. With such a legislative body the best interests of medical schools are safe, because it is to be presumed that the ends which each have in view are the same, to wit., the interests of medical education. The authority of a body thus constituted will afford protection against local factions, whose jealousy prompts hostility to medical institutions, and cannot fail to do much toward securing for those schools which recognise it, general confidence and respect, since its behests are the expressed wishes of the profession at large. Entertaining these views we shall rejoice to see the position taken by other institutions, which has been taken by the Medical Department of the University of Pennsylvania, and, also, virtually, by the College of Physicians of the city of New York. We anticipate that this will be done, and that it will conduce to the interests equally of the institutions and the whole profession.

With these prefatory remarks we present to our readers the following extracts from the report which has suggested them:—

"Since the last annual communication of the Faculty, an occurrence has taken place of great interest to the Medical Profession, and likely to exert no little influence over the future character of medical instruction in this country. A Convention of Physicians, representing medical bodies in almost all sections of the Union, assembled in Philadelphia in May last, to take into consideration the various interests of the profession, and to adopt measures calculated to sustain and elevate its character and usefulness. It is believed that, in relation to its numbers, and the standing of its individual members, the late convention has never been equalled by any assemblage of medical men upon this continent. The recommendations of such a body are entitled to the highest respect; and, though it may not be practicable to carry them immediately into full effect, yet, as they have the general good only in view, it would appear to be incumbent on all to enter into their spirit, and by cordial efforts to prepare the way for the ultimate attainment of their objects. The Faculty recognise this obligation, and propose to act in accordance with it.

A prominent measure among those recommended by the Convention is the prolongation of the annual course of scholastic medical instruction to six months. The Faculty have long been sensible of the evils of the limited period generally allotted to their courses by the schools. It was adopted in the infancy of medical teaching in this country, and at a period when our science was much less expanded than at present. The rapid growth of medicine in almost all its brances, and especially the increased attention to the important subject of demonstration, have greatly multiplied the number of lectures; and, independently of these causes, the advancing wealth and intelligence of the country have called for a corresponding expansion of the means of knowledge in relation to one of its most important interests. Yet it has been deemed necessary to crowd this increased amount of instruction within the limits of time originally fixed; and the consequence has been that the lectures follow each other in such rapid succession, as to task unduly both the mental and physical powers of the pupil. It is only by a painful concentration of the attention, and the devotion of their whole time to study during the allotted period, to the exclusion of necessary rest and exercise, that even the most advanced students can follow satisfactorily the different courses; and the younger must necessarily be content with an imperfect understanding of the subject. There cannot be the least doubt, that, by spreading the same amount of insruction over a larger space of time, the student would gain a more than proportional increase of knowledge, with less strain upon his faculties, and less injury to his health.

Influenced by considerations of this kind, the Medical Faculty attempted, so long since as the year 1836, to lengthen the course trom four to five months, by continuing the lectures from the ordinary time of beginning in the first week of November, to the last of March; while they instituted preliminary lectures during the month of October; so that virtually the course of instruction was extended through six months, for all those who might find it convenient to attend. The attempt, however, met with only partial success. The preliminary lectures have been continued with greater or less regularity to the present time, and have been attended by classes varying from 100 to 250 in number. But, as the example of the University was not followed by other schools, which contiuued to terminate their sessions at the close of February, it was found impossible to retain the students of the first course throughout the month of March; and the Faculty deemed it expedient to close their lectures in the middle of March, thus gaining two weeks upon the original length of the regular session, instead of the four weeks at first contemplated.

With the support of the late Convention, and with the presumed countenance of the profession at large, the Faculty are disposed to make a fresh effort to accomplish this very desirable object. In Europe, the necessity of a period of scholastic instruction longer than the one habitual in this country, is universally recognised; and it is believed that in no distinguished medical school abroad is the annual session shorter than six months. There is certainly nothing in the condition of the United States which can justify their lingering reluctantly behind the communities of Europe in the means of promoting the knowledge of so important a science as that of medicine. There is as much wealth and intelligence diffused among the great masses of people in this country as abroad; and the pecuniary means of those who study for the medical profession, are probably on the average greater than those of the European student. Why, therefore, should we delay in widening our limits of instruction till they equal those of communities not more advanced in other respects? Will the world, will our posterity justify us in remaining voluntarily in a position of inferiority, which, independently of other weighty considerations, must affect the credit of our beloved country in contemporary estimation and in history?

It is admitted by the Faculty that, in adopting the present plan, they do so at the risk of diminishing the numbers of the class, and consequently their own emoluments. But they look to the profession for support in this attempt to elevate its character by improving the education of those about to enter it. They felt it incumbent upon them, as representing the oldest medical school in the country, to come forward and offer to the profession this opportunity of carrying into effect what appears to be its own cherished wish, and is certainly in itself highly desirable. Should the attempt prove successful, other schools will probably come into the measure; and we may thus hope to witness a universal improvement of medical education in the United States. Should it fail, the Faculty will at least have the consolation of having acted in accordance with their sense of duty.

In extending the period of scholastic instruction, the Faculty have no wish to encroach on the rights of the private preceptor. They fully and cordially recognise the importance of office instruction, and deem the opportunities enjoyed by the student of acquiring practical knowledge, by visiting the sick, and preparing medicines for their use, under the immediate eye of his preceptor, as of the utmost value in the scheme of medical tuition. They do not think that an addition of four weeks to each winter session will be coosidered an unduly encroaching upon the period of private study; and the preceptor will probably find, that the increased ability of the student to make a profitable use of the interval between the sessions, will much more than compensate for the slight diminution of that interval.

In relation to the qualifications for graduation, the Faculty do not perceive that they can at present advantageously extend their system. It has always been their wish, that the honors of the Institution with which they are connected, should be more than nominal; that the degree of Doctor of Medicine in the University should be a true testimonial of respectable medical attainment. To this end their final examinations have always been directed. Without exacting of the student an amount of knowledge beyond what might be reasonably expected from ordinary industry applied during the regular period of study, they have aimed to exclude ignorance from the honors of the school; and they can confidently appeal to the general standing of the graduates as evidence of their success. As a particular instance, they may perhaps be permitted, without the imputation of undue boasting, to allude to the number of the graduates of the University who have distinguished themselves as public teachers, in the numerous medical schools which have sprung into existence during the last thirty years. That in time, under the influence of enlightened public sentiment, the standard of graduation may be still further elevated, is the sincere hope, and will be the honest endeavor of the Faculty, should their present efforts meet with the co-operation of the profession and the schools.

In compliance with a resolution of the recent Convention, the Faculty strongly recommend to the young gentlemen who may propose attending their instructions during the ensuing course, to bring with them letters from their several preceptors to the Dean of the Faculty, stating their age, the period at which they began their professional studies, and the general character of their preliminary attainments, so far as these may be known to the teacher.

In closing this communication, the Faculty would once more appeal to the public spirit of the profession. Of themselves, they can do no more than offer the means for an expanded system of medical instruction. Upon the profession itself, must depend the issue. At no time has there been a fairer opening for a combined effort to place the study and practice of medicine in that elevated position to which their nature and objects would appear to entitle them. Should this effort not be made, or should it fail from a want of hearty and united co-operation, the fear is that a condition of mediocrity will be fixed upon this great interest of our country, and that for a long series of years, the friends of medicine, instead of working with the cheering prospect of a continued advance before them, will find themselves engaged in the irksome and discouraging task of withstanding the deteriorating tendencies of a mere business competition.

